# Association between limb alignment and patient-reported outcomes after total knee arthroplasty using an implant that reproduces anatomical geometry

**DOI:** 10.1186/s13018-018-1030-8

**Published:** 2018-12-17

**Authors:** Arata Nakajima, Masato Sonobe, Yorikazu Akatsu, Yasuchika Aoki, Hiroshi Takahashi, Toru Suguro, Koichi Nakagawa

**Affiliations:** 1 Department of Orthopaedic Surgery, Toho University Sakura Medical Center, 564-1 Shimoshizu, Sakura-shi, Chiba 285-8741 Japan; 2 Department of General Medical Sciences, Graduate School of Medicine, Chiba University, 1-8-1 Inohana, Chuo-ku, Chiba-shi, Chiba 260-8677 Japan; 3 Department of Orthopaedic Surgery, Eastern Chiba Medical Center, 3-6-2 Okayamadai, Togane-shi, Chiba 283-8686 Japan; 4 Japan Research Institute of Artificial Joint, 725-1 Sugo, Kisarazu-shi, Chiba 292-0036 Japan

**Keywords:** Total knee arthroplasty (TKA), Anatomical geometry, Limb alignment, Kinematic alignment, Patient-reported outcomes (PROs)

## Abstract

**Background:**

A kinematically aligned (KA) total knee arthroplasty (TKA) is expected to improve patient satisfaction, but its effect remains controversial. We investigated differences in patient-reported outcomes (PROs) between KA and non-KA TKAs using an implant that reproduces anatomical geometry.

**Methods:**

TKAs for varus deformity were performed in consecutive 129 patients (149 knees) via a measured resection technique with conventional instruments. The femorotibial angle (FTA), hip-knee-ankle angle (HKAA), and the angle between the joint line and the line perpendicular to the mechanical axis (AJLMA) were measured postoperatively (mean 13.6 months), and an AJLMA of ≥ 2° was defined as kinematic alignment. Patients were assigned to two or three alignment categories in each measurement method, and the Knee Society Scores (KSS) and Japanese Knee Injury and Osteoarthritis Outcome Scores (J-KOOS) was compared among the groups.

**Results:**

For patients assessed by FTA, an ADL-related J-KOOS subscale (J-KOOS-A) showed a significant difference between valgus and varus outliers (*p* < 0.05). When assessed by HKAA, neither the KSS nor J-KOOS subscales were significantly different among groups. When assessed by AJLMA, J-KOOS-A was significantly different between groups, and a group for AJLMA of ≥ 2° had higher scores than a group for AJLMA of < 2° (95% CI 0.323–7.763; *p* < 0.05).

**Conclusions:**

Patients with an AJLMA of ≥ 2° reported significantly higher patient’s satisfaction regarding ADL. This suggests the importance of restoration of the physiological joint line which can be achieved via KA TKAs.

## Background

Postoperative restoration of a neutral limb alignment to preserve knee function and longevity has been the primary goal of conventional total knee arthroplasty (TKA) over the past two decades [1, 2]. Conventional TKAs have relieved patients’ symptoms of pain and corrected deformities, resulting in improvements in the activities of daily living (ADL). However, in general, patients’ satisfaction with TKAs is not as favorable as it is for total hip arthroplasties [3–5], generating the need for improved surgical techniques and new technological developments.

Recently, kinematically aligned (KA) TKAs were introduced by Bellemans [6]. With KA TKAs, the femoral and tibial components are implanted with mild varus limb alignment, relative to neutral alignment, in order to restore the physiological joint line to a pre-arthritic state. Whether KA TKAs are superior to the mechanically aligned (MA) TKAs based on patients’ postoperative satisfaction has been an ongoing point of debate [7–9]. Furthermore, little information is available in terms of the postoperative association between the limb alignment and patient-reported outcomes (PROs) in KA TKAs.

**Fig. 1:**
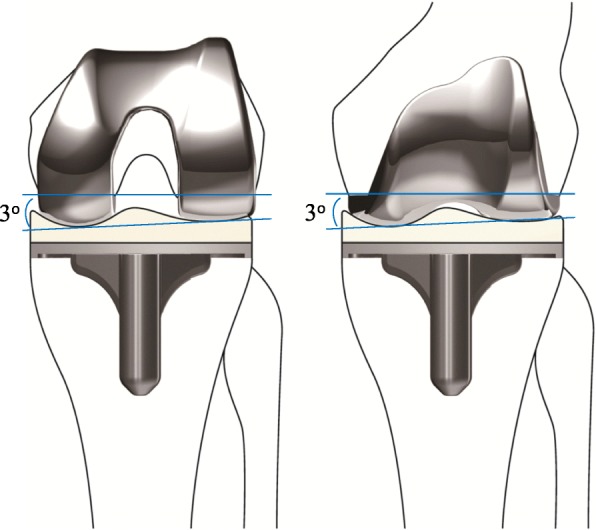
The FINE total knee. The femoral condyle has an asymmetric shape and femorotibial joint line with an oblique 3° angle both in coronal (left) and axial (right) planes which is incorporated into the implant design. The medial surface of the polyethylene insert has a convex curve while the lateral surface possesses a flat surface. FINE reproduces anatomical geometry by conducting osteotomy perpendicular to the mechanical axis

The FINE total knee (Teijin-Nakashima Medical, Okayama, Japan) has unique design features, including a femorotibial joint line with an oblique 3° angle (Fig. 1). This feature enables the implant to reproduce anatomical geometry and allows the osteotomy to be performed perpendicular to the mechanical axis. The FINE total knee is also designed to guide internal movements of the tibia via medial pivotal rotation, thus permitting deeper flexion of the knee to better match the lifestyle needs of Japanese populations [10]. The medial surface of the polyethylene insert has a convex curve which is designed to increase the rate of conformity to the femoral component, thereby enhancing internal rotation of the tibia. Conversely, the lateral surface has a flat surface which has been designed to allow femoral rollback, thereby enhancing internal rotation of the tibia via medial pivotal motion [10]. Hence, the design concepts of FINE total knee facilitate to obtain kinematic alignment via conventional osteotomy performed for MA TKAs.

The aim of the present study was to investigate, retrospectively, whether there are differences in the postoperative patient-reported outcomes (PROs) including Knee Society Score (KSS) and Knee Injury and Osteoarthritis Outcome Score (KOOS) in different alignment categories for patients undergoing TKAs using the FINE total knee. We hypothesized that the KSS would be similar among the groups, but that patients with kinematic alignment would have a higher KOOS compared with those without it.

## Patients and methods

### Patients

A total of consecutive 129 patients (24 males and 105 females) underwent primary TKAs (149 knees) for varus knees resulting from osteoarthritis or rheumatoid arthritis at our institution between August 2013 and January 2016 and were included in this study. The exclusion criteria included valgus deformity, occurrence of fractures in lower limbs receiving TKAs, and deterioration of dementia during the follow-up period. Preoperative patient demographics and knee physical function indicators such as deformities, range of movement (ROM), and KSS are shown in Table 1.

**Table 1:** Patients’ demographics, preoperative deformities, ROM, and KSS

Number of patients (male/female)	129 (24/105)
Implant type (CR/PS)	115/34
Age, years old	73.8 ± 8.1
BMI, kg/m^2^	26.5 ± 4.6
Follow-up period, months (range)	13.6 ± 2.6 (12–24)
FTA, degrees	185.0 ± 5.4
HKAA, degrees	13.1 ± 6.4
Extension, degrees	− 9.3 ± 11.0
Flexion, degrees	120.6 ± 17.1
ROM, degrees	111.3 ± 24.3
KSS-KS	44.5 ± 13.0
KSS-FS	36.9 ± 20.1
KSS-Combined	81.4 ± 27.6

*CR* cruciate-retaining, *PS* posterior cruciate ligament-substituting, *BMI* body mass index, *FTA* femorotibial angle, *HKAA* hip-knee-ankle angle, *ROM* range of movement. Data are expressed as a mean ± SD

### Surgical procedures

All implants used in this study were FINE total knee, of which 115 were the cruciate-retaining type and 34 were the posterior ligament-substituting type. Surgeries were performed using a measured resection technique and conventional instruments, that is, the distal femoral osteotomy was conducted perpendicular to the mechanical axis and the posterior condyle was osteotomized parallel to the surgical epicondylar axis; a tibial osteotomy was subsequently conducted perpendicular to the anatomical axis of the tibia. Following osteotomy, adjustments for soft tissue balancing were performed before the implants were fixed to the bone with cement.

### Radiographic examinations

**Fig. 2:**
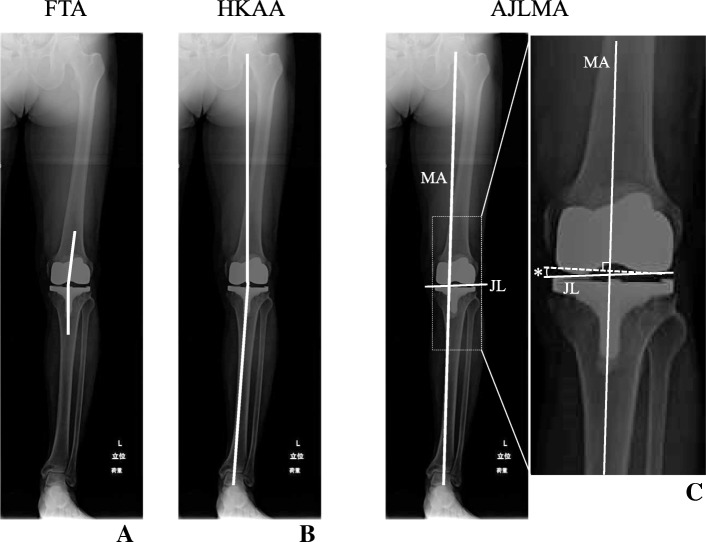
The femorotibial angle (FTA) is the angle between the anatomical axes of the femur and tibia (**a**), the hip-knee-ankle angle (HKAA) is the angle between the mechanical axes of the femur and tibia (**b**), and AJLMA is the angle between the joint-line and the line perpendicular to the mechanical axis (**c** right, asterisk). A dotted box in the left is magnified in the right. MA, mechanical axis; JL, joint line

At the time of follow-up, we measured and assigned categories for the coronal alignment of lower limbs using different evaluations for the angles assessed; the femorotibial angle (FTA) was the angle between the anatomical axes of the femur and tibia (Fig. 2a), and the hip-knee-ankle angle (HKAA) was the angle between the mechanical axes of the femur and tibia (Fig. 2b). In addition to these radiographic examinations, we measured the angle between the joint line and the line perpendicular to the mechanical axis (AJLMA) to investigate whether the implants were set in kinematic alignment (Fig. 2c right, asterisk). FTAs and HKAAs were evaluated and considered in-range if the angle was 173–177° and − 3 to 3°, respectively, whereas values outside of this range were categorized as either varus or valgus outliers in reference to the previous publication [11]. For the AJLMA category, we assigned patients into two groups: group A included patients with an AJLMA of < 2° and group B included patients with an AJLMA of ≥ 2°. Group B, but not group A, was defined as kinematic alignment.

### PROs

We used the KSS as an objective evaluation of knee function, which consists of the knee score (KSS-KS), the function score (KSS-FS), and their combined score (KSS-combined). In addition to the KSS, we examined the Japanese KOOS (J-KOOS), an instrument of confirmed validity and reliability for PROs based on its cross-cultural adaptation [12]. The KOOS consisted of a total of 42 knee-related items, and each item was scored from 0 to 4. Five KOOS subscales, including symptoms (KOOS-S), pain (KOOS-P), ADL (KOOS-A), sports/recreation (KOOS-SP), and quality of life (KOOS-Q), were converted to 100 points [13]. This study was approved by the institutional review board at our institution (application number: S17012). All activities were performed in accordance with the ethical standards set forth in the Declaration of Helsinki.

### Statistical analysis

The reliability of each radiographic measurement was assessed using intraclass correlation coefficients. All radiographic measurements in this study showed good reliability (all values > 0.8). We compared the KSS and J-KOOS among different alignment categories of patients assessed by FTA, HKAA, and AJLMA. Results were expressed as the mean ± standard deviation (SD). Comparisons between patients assessed by FTA and HKAA were performed by one-factor ANOVA, and those between groups A and B for AJLMA assessments were performed using a *t* test. A statistical power analysis was performed prior the study; based on a prespecified significance level of *α* < 0.05 and by assuming a medium effect size (= 0.5), the power required was estimated to be 0.8 by using G*Power 3. The estimated sample size was 64 patients. Data analyses were performed using SPSS software, version 21 (SPSS Inc., Chicago, IL, USA), and *p* values of < 0.05 were considered to be statistically significant.

## Results

Postoperative limb alignment, ROM, KSS, and J-KOOS are shown in Table 2. Overall, average KSS-KS, KSS-FS, and KSS-combined scores were 96.1, 74.1, and 170.2, respectively. The J-KOOS-S, J-KOOS-P, J-KOOS-A, J-KOOS-SP, and J-KOOS-Q were 80.3, 87.1, 85.2, 48.6, and 62.0, respectively.

**Table 2:** Postoperative limb alignment, ROM, KSS, and J-KOOS

FTA, degrees	175.1 ± 2.2
HKAA, degrees	2.52 ± 3.43
AJLMA, degrees	1.44 ± 1.59
Extension, degrees	− 0.95 ± 3.32
Flexion, degrees	122.1 ± 14.2
ROM, degrees	121.2 ± 15.5
Increase in ROM, degrees	9.7 ± 18.6
KSS-KS	96.1 ± 5.0
KSS-FS	74.1 ± 20.3
KSS-Combined	170.2 ± 22.6
J-KOOS-S	80.3 ± 14.2
J-KOOS-P	87.1 ± 13.4
J-KOOS-A	85.2 ± 11.6
J-KOOS-SP	48.6 ± 28.5
J-KOOS-Q	62.0 ± 21.9

*FTA* femorotibial angle, *HKAA* hip-knee-ankle angle, *AJLMA* angle between the joint-line and the line perpendicular to the mechanical axis, *ROM* range of movement. Data are expressed as a mean ± SD

**Fig. 3:**
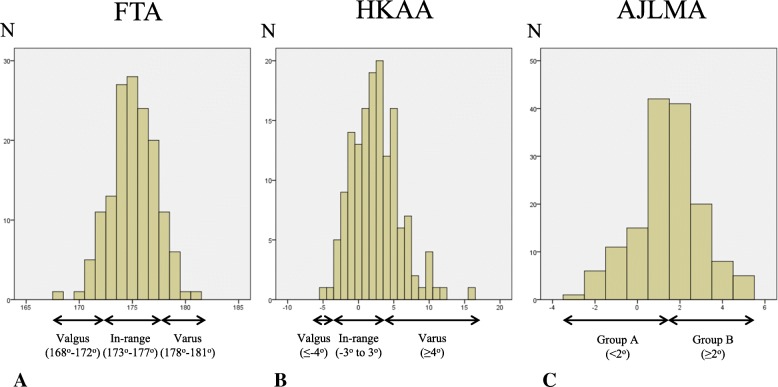
**a** When patients were assessed by FTA postoperatively, 74.5% were in-range (173–177°). Both valgus (168–172°) and varus (178–181°) outliers were 12.8%. **b** When patients were assessed by HKAA, 64.4% were in-range (− 3 to 3°), and 1.3% were valgus (≤ 4°) outliers and 34.2% were varus (≥ 4°) outliers. **c** For the AJLMA assessment, group A included 75 knees with an AJLMA of < 2° and group B included 74 knees with an AJLMA of ≥ 2°

When patients were postoperatively assessed by FTA, 74.5% of them were in-range (173–177°). Both valgus (168–172°) and varus (178–181°) outliers were 12.8% (Fig. 3a). There were no significant differences among groups for the KSS-KS, KSS-FS, and KSS-combined as well as the J-KOOS-S, J-KOOS-P, J-KOOS-SP, and J-KOOS-Q; however, there was a significant difference (*p* < 0.05) in the J-KOOS-A between valgus and varus outliers (Table 3, left).

**Table 3:** Postoperative KSS and J-KOOS in the alignment categories for FTA, HKAA, and AJLMA

	FTA				HKAA				AJLMA			
	Varus (*n* = 19)	In-range (*n* = 111)	Valgus (*n* = 19)	*p*	Varus (*n* = 51)	In-range (*n* = 96)	Valgus (*n* = 2)	*p*	< 2 (group A, *n* = 75)	≥ 2 (group B, *n* = 74)	Difference (95% CI)	*p*
KSS-KS	96.4 ± 4.2	96.1 ± 5.1	95.9 ± 4.8	0.967	96.5 ± 4.7	95.9 ± 5.1	100.0 ± 0.0	0.425	95.8 ± 5.3	96.4 ± 4.6	0.582 (− 1.059 to 2.222)	0.484
KSS-FS	81.6 ± 13.3	73.5 ± 20.6	69.4 ± 23.4	0.164	74.4 ± 17.9	73.7 ± 21.8	80.0 ± 0.0	0.902	71.0 ± 22.4	77.0 ± 17.8	5.986 (− 0.679 to 12.651)	0.078
KSS-Combined	178.0 ± 14.3	169.6 ± 23.0	165.4 ± 26.2	0.211	170.8 ± 20.2	169.6 ± 24.1	180.0 ± 0.0	0.792	166.8 ± 24.8	173.4 ± 20.0	6.64 (− 0.783 to 14.062)	0.079
J-KOOS-S	81.2 ± 15.7	80.7 ± 13.8	76.7 ± 15.5	0.508	80.0 ± 14.4	80.1 ± 14.1	94.5 ± 2.1	0.363	79.9 ± 13.5	80.6 ± 15.0	0.715 (− 3.895 to 5.326)	0.760
J-KOOS-P	87.1 ± 17.7	87.7 ± 12.5	83.9 ± 13.9	0.521	86.9 ± 14.7	87.1 ± 12.8	97.0 ± 0.0	0.581	87.3 ± 13.1	87.0 ± 13.8	0.361(− 4.718 to 3.997)	0.870
J-KOOS-A	89.7 ± 9.7	85.2 ± 10.9	80.4 ± 15.5	0.046*	84.8 ± 11.6	85.1 ± 11.7	98.5 ± 2.1	0.261	83.2 ± 12.3	87.2 ± 10.6	4.043 (0.323 to 7.763)	0.033*
J-KOOS-SP	52.6 ± 33.8	48.0 ± 28.3	47.6 ± 25.5	0.803	50.0 ± 29.5	47.3 ± 28.2	75.0 ± 0.0	0.362	46.0 ± 28.5	51.2 ± 28.5	5.162 (− 4.075 to 14.4)	0.271
J-KOOS-Q	65.8 ± 24.6	62.2 ± 21.5	57. 2 ± 21.5	0.473	61.7 ± 25.4	61.9 ± 20.1	75.0 ± 0.0	0.701	60.2 ± 20.5	63.9 ± 23.2	3.665 (− 3.415 to 10.744)	0.308

*FTA* femorotibial angle. FTAs were evaluated and considered in-range if the angle was 173–177°, whereas values outside of this range were categorized as either varus or valgus outliers. *HKAA* hip-knee-ankle angle. HKAAs were evaluated and considered in-range if the angle was 0 ± 3^o^, whereas values outside of this range were categorized as either varus or valgus outliers. *AJLMA* angle between the joint line and the line perpendicular to the mechanical axis. Group A included patients with an AJLMA of < 2° and group B included patients with an AJLMA of ≥ 2°. Group B, but not group A, was defined as kinematic alignment. Data are expressed as mean ± SD. *Significantly different (*p* < 0.05)

When patients were assessed by HKAA, 64.4% were in-range (− 3 to 3°). Valgus (≤ 4°) and varus (≥ 4°) outliers were 1.3% and 34.2%, respectively (Fig. 3b). There were no significant differences in KSS-KS, KSS-FS, and KSS-combined among the groups. Furthermore, none of the J-KOOS subscales were significantly different among groups (Table 3, middle).

For patients assessed by AJLMA, 50.3% fit into group A (< 2°) and 49.7% fit into group B (≥ 2°) (Fig. 3c). There were no significant differences in the KSS-KS, KSS-FS, and KSS-combined between groups A and B. Of the J-KOOS subscales, J-KOOS-A alone was significantly different between groups, with group B demonstrating significantly higher scores than group A (Table 3, right; 95% CI 0.323–7.763, *p* < 0.05).

## Discussion

The most important findings of the present study were that patients with a postoperative AJLMA of ≥ 2° scored significantly higher in J-KOOS-A than those with a postoperative AJLMA of < 2°, and there were no significant differences in other J-KOOS subscales and the KSS between groups. These results suggest that reproducing the physiological joint line is important in order to reach higher levels in the ADL after TKA.

The FINE total knee is a unique prosthesis that has an oblique 3° angle in the medial femoral condyle and the polyethylene insert [10]. This design could have advantages considering implants are set in kinematic alignment by performing the osteotomy in neutral alignment. Most implants that are currently used in the world have symmetrical medial and lateral femoral condyles designed to be implanted perpendicular to the mechanical axis. Therefore, in order to set such implants in kinematic alignment, surgeons have to perform a distal femoral osteotomy with more valgus alignment than normal. Furthermore, the posterior condyle has to be osteotomized with mild internal rotation relative to normal. These KA TKAs, using conventional implants, can be a cause of concern in relation to long-term clinical results, loosening of implants, or longevity of the polyethylene inserts [8, 9, 14, 15]. Ishikawa et al. have reported that patellofemoral and tibiofemoral contact stresses were increased in KA TKAs when the femoral component was implanted in a more valgus and internally rotated position, and with the tibial component in a more varus and internally rotated position [15].

It is still controversial as to whether KA TKAs are superior to MA TKAs in terms of patient satisfaction. Dossett et al. have reported that the use of a kinematic alignment technique provided better pain relief and restored better function and range of movement than a mechanical alignment technique [7]. Conversely, Waterson et al. [8] and Young et al. [9] have reported that there were no significant differences in the KOOS, KSS, SF-36, EQ-5D, 2-min walk, Timed Up and Go (TUG), Oxford Knee Score (OKS), WOMAC, and Forgotten Joint Scores. Thus, randomized control trials (RCTs) so far have not revealed that the KA TKA is superior to the MA TKA in terms of PROs. However, in these RCTs, authors compared postoperative clinical results simply based on the difference in surgical procedures (i.e., KA TKAs versus MA TKAs) and not by differences in the limb alignment (i.e., in-range versus varus or valgus outliers and kinematic versus non-kinematic alignments). Thus, it is questionable as to whether the implants were actually set in kinematic alignment, even for KA TKAs. Therefore, in the present study, we assigned patients into two groups for the assessment of the AJLMA, such that the effect of “true” kinematic alignment could be discerned on the basis of PROs to clarify the relationship.

Hutt et al. have reported the postoperative joint line orientation angle (JLOA), that is, the angle between the joint line and the line parallel to the floor [16], as a parameter for kinematic alignment. It is still controversial as to what constitutes the best radiographic parameter for kinematic alignment, but in the present study, we set the AJLMA as a parameter for this. AJLMA could be a useful parameter to evaluate kinematic alignment since it is not affected by the foot position of subjects. In this study, patients were categorized into two groups on the basis of an AJLMA ≥ 2° or an AJLMA < 2°, and we set the AJLMA cutoff value of 2° for the kinematic alignment. This is because the proportion of patients between groups could be optimized (*n* = 74, AJLMA ≥ 2° versus *n* = 75, AJLMA < 2°). Patients with an AJLMA ≥ 2° showed significantly higher scores for J-KOOS-A relative to those with an AJLMA of < 2°, indicating the importance of a medial inclination of the joint line which can lead to better improvements in the ADL of patients.

The Knee Society Function Score (KS-FS), also commonly used, has been validated in numerous studies as a reliable way to evaluate postoperative TKA outcomes [17, 18]; however, PROs obtained via the KS-FS may not reflect changes in outcomes over time as responsively as other instruments such as WOMAC or SF-36 [18–21]. While the KOOS is rarely employed for the evaluation of TKA, it has been shown that the KOOS has a higher responsiveness and a lower ceiling effect, making it a superior outcome tool relative to the KS-FS when evaluating the outcomes of TKA patients [22]. Relative to the WOMAC or OKS, the KOOS has more items for ADL that are related to knee function and is considered to be most important to patients receiving TKAs. In this study, we showed significant differences in J-KOOS-A between KA and non-KA TKAs. Recent reports regarding the relationship between coronal alignment and clinical results have shown no significant effects of postoperative coronal alignment on the KSS [23, 24]. Howell et al. showed that there were no significant differences in the OKS and WOMAC in any alignment categories for KA TKAs during a follow-up period of 31–34 months [11]. Because the OKS has only 12 items that are not specific to ADL, and the WOMAC is not a knee-specific outcome score, they might have failed to detect significant differences between the KA and non-KA TKAs.

In this study, using an implant that reproduces anatomical geometry, we performed TKAs via a method that sets the implants in neutral alignment, which we expected would automatically produce kinematic alignment. However, approximately half of the implants were not KA. This is partial because the osteotomy was not performed correctly as planned, but suggests that appropriate soft tissue balancing could also be required to achieve the kinematic alignment.

This study has some limitations. First, a follow-up period is short, and mid to long-term follow-up will be required to evaluate if the kinematic alignment can give better patient-reported outcomes. Second, 23% of the patients received a PS implant, which may reproduce a different type of motion from a CR implant and eventually affect PROs. Third, we defined AJLMA to evaluate the kinematic alignment and assigned patients into two groups as a cutoff value of 2°. Since AJLMA is not a validated angle, other measurement methods such as JLOA might be better to evaluate the kinematic alignment.

Despite limitations such as these, surgeons can expect better reports of ADL from patients whose implants were set in kinematic alignment. Although a half of the knees were not able to achieve the kinematic alignment in this study, an implant that reproduces anatomical geometry could be useful on the basis of its potential to achieve kinematic alignment by allowing a conventional osteotomy to be performed. This in turn may also be beneficial in terms of reducing contact stress of the polyethylene insert which can contribute to the longevity of the implants.

## Conclusions

The present study showed that patients with an AJLMA of ≥ 2° had significantly higher patient’s satisfaction in ADL than those with an AJLMA of < 2°. This suggests the importance of restoration of the physiological joint line which can be achieved via KA TKAs. To evaluate the superiority of the kinematic alignment, mid to long-term clinical results regarding PROs will be required.

## Abbreviations

AJLMA: Angle between the joint line and the line perpendicular to the mechanical axis
FTA: Femorotibial angle
HKAA: Hip-knee-ankle angle
J-KOOS: Japanese Knee Injury and Osteoarthritis Outcome Scores
KA: Kinematically aligned
KSS: Knee Society Scores
MA: Mechanically aligned
PROs: Patient-reported outcomes
TKA: Total knee arthroplasty
